# Electrophysiological evaluation and 18-month follow-up of two regimens with aflibercept for neovascular age-related macular degeneration

**DOI:** 10.1007/s10633-021-09863-7

**Published:** 2022-02-26

**Authors:** Marion Schroeder, Ulrika Kjellström, Monica Lövestam-Adrian

**Affiliations:** 1 Department of Clinical Sciences Lund, Ophthalmology, Lund University, Skane University Hospital, Lund, Sweden; 2 Department of Ophthalmology, Lund University, 221 85 Lund, Sweden

**Keywords:** Neovascular age-related macular degeneration, Full-field electroretinography, Multifocal electroretinography, Treat-and-extend, Aflibercept

## Abstract

**Purpose:**

To compare two aflibercept treatment regimens and the electrophysiological outcome concerning cone and rod function in age-related macular degeneration (nAMD) over 18 months.

**Methods:**

41 patients with treatment-naïve nAMD were randomized 1:1 to either arm 1 or 2. Arm 1 received three consecutive monthly aflibercept injections, followed by bimonthly treatment until week 52. Thereafter, a treat-and-extend (TAE) regimen was applied. Arm 2 was treated according to a TAE protocol throughout the 18-month follow-up. We assessed visual acuity (VA), central retinal thickness (CRT), injection rate and interval, and evaluated cone and rod function with full-field and multifocal electroretinography (ffERG, mERG).

**Results:**

There were no statistically significant differences in mean baseline VA, lesion type, age, gender, or symptom duration between the two arms. During the 18-month follow-up, mean VA improved in arm 1 (*n* = 19) from 63.5 ± 10.5 to 69.1 ± 9.2 letters; *p* = 0.098; and in arm 2 (*n* = 20) from 66.8 ± 13.6 to 73.9 ± 9.0 letters; *p* = .002. In both arms, mean CRT was significantly reduced; *p* < 0.000. At month 18, we found no significant difference in the number of injections or injection intervals between groups. Arm 1 had received 11.3 ± 1.7 injections vs. 10.9 ± 2.0 in arm 2. The mean injection interval was 9.2 ± 3.4 weeks vs. 9.5 ± 3.1, with 52% (*n* = 10) on the maximum 12-week interval in arm 1, and 50% (*n* = 10) in arm 2. The combined rod-cone a-wave amplitude significantly decreased over time; *p* = 0.043. The isolated rod b-wave amplitude showed a statistically significant decline; *p* = 0.026. The overall mERG amplitude and implicit time remained unchanged over time; *p* = 0.878 vs. *p* = 0.922. The central ring 1 mERG amplitude improved; *p* = 0.041, with an unaffected implicit time.

**Conclusions:**

After 18 months, both treatments arms have received a similar number of injections at comparable intervals. Electrophysiological evaluation shows no signs of toxicity concerning cone function. But ffERGs for the combined and isolated rod response have declined, possibly reflecting either toxic effects of the drug to rods or the natural course of the disease itself.

## Introduction

Age-related macular degeneration (AMD) presents a leading cause of severe visual loss in the elderly population in developed countries. Approximately 8–12% of people with AMD develop neovascular AMD (nAMD) [1, 2] that requires treatment with intravitreal injections to inhibit vascular endothelial growth factor (VEGF). One of the available drugs is aflibercept, a fusion protein with VEGF receptors 1 (VEGFR1) and 2 (VEGFR2), key domains binding VEGF-A, VEGF- B, and placental growth factor (PIGF). The recommended dose is 2 mg aflibercept, equivalent to 50 µl. Treatment is initiated with one injection per month for three consecutive doses followed by bimonthly injections until the end of year one [3].

Generally, there are different treatment regimens, such as monthly evaluation with as-needed injections secondary to signs of activity; pro re nata (PRN). Another widely approved treatment regimen is treat-and-extend (TAE), a more proactive regimen with an injection administered at each visit. After a loading dose, the treatment interval is extended if there are no longer signs of activity, and reduced in case of recurrence. TAE is considered equally effective as monthly treatment and superior to PRN by reducing the number of injections and visits, and improving visual acuity outcome and persistence of treatment [4].

Special concerns relate to the long-term effects on the retinal cells when repeatedly injected with anti-VEGF agents. Different electrophysiological evaluation methods have been established to measure photoreceptor function. Full-field electroretinography (ffERG) is used to evaluate the response of rods and cones of the total retina. Multifocal electroretinography (mERG) measures the cone function of the central retina. In vitro, aflibercept shows no sustained short-term toxic effect on photoreceptors in the outer retina layers on the electroretinogram [5]. Previous results of mERG measurements after intravitreal anti-VEGF treatment have shown a short-term improvement in macular function [6]. In contrast, other studies report no functional change during intravitreal treatment [7] or find rod and cone dysfunction, some with preceding early-onset rod-mediated dysfunction in early and intermediate AMD [6, 8–10].

In this prospective study, we compared two aflibercept treatment regimens in patients with treatment-naïve nAMD. Our patients received either TAE from the start or a fixed regimen with a loading dose consisting of three consecutive monthly injections, followed by a bimonthly treatment interval until week 52, after which patients switched to the TAE protocol until the end of follow-up at month 18. Therapeutic effects of intravitreal anti-VEGF treatment was assessed with ff- and mERG over time, and the outcome of the two treatment regimens was compared concerning best corrected visual acuity (BCVA), central retinal thickness (CRT), injection rate and injection interval.

## Methods

### Study design

Our study was an 18-month, prospective, randomized, non-controlled pilot study with aflibercept. The study was approved by the institutional review board in Lund and conducted in conformity with the Declaration of Helsinki. Patients signed an informed consent prior to randomization.

We included 41 eyes of 41 patients with treatment-naïve nAMD. Inclusion criteria were macular neovascularization types 1–3, divided into occult, minimally classic or predominantly classic lesions, and retinal angiomatous proliferations (RAP) with symptom duration of ≤ 6 months, and visual acuity ≥ 35 letters. The patients were consecutively recruited from the county of Skane in Sweden between February 2015 and July 2016 and were 1:1 randomized before treatment start.

### Treatment protocols

Patients with a diagnosis of nAMD were randomized to one of two treatment arms. Arm 1, per label regimen, comprised a loading dose with three monthly injections, followed by injections every eight weeks (q8) until week 52. Thereafter, patients were switched to a treat-and-extend regimen. That meant an extension of the interval by 2 weeks if the macula appeared to be dry or with a stable subretinal fluid layer on optic coherence tomography (OCT). Confirmed signs of activity, as intraretinal macular edema, increasing subretinal fluid, or a new, small hemorrhage lead to a reduction in the treatment interval by at least 2 weeks, with 4 weeks as the shortest possible interval, until the macula was dry or with a stable subretinal fluid layer on OCT at two consecutive visits. In case of a modest or larger hemorrhage, the interval was to be reduced to 4 weeks. Patients in arm 2, TAE, started with an injection interval of 4 weeks and could be extended to 6 weeks already after injection number two if presenting dry on OCT. Otherwise, the group had the same TAE change of interval criteria as above. The maximum reachable interval of treatment for arms 1 and 2 was 12 weeks.

Of the total of 41 patients, 21 patients were randomized to arm 1, and 20 patients to arm 2. Two patients in arm 1 did not complete the 18-month follow-up. One patient chose to withdraw from the trial because of low visual acuity after an early retinal pigment epithelial tear. The other patient switched to ranibizumab after a non-infectious endophthalmitis.

### Ophthalmological examination

At the baseline visit, 1–7 days before the first intravitreal treatment, and at months 6 and 18, always 1 month, ± 1 week, after the last given injection, patients underwent best corrected visual acuity (BCVA) assessment using the Early Treatment Diabetic Retinopathy Study (ETDRS) scale, slit-lamp biomicroscopy, tonometry, dilated funduscopy, optical coherence tomography (OCT), full-field electroretinography (ffERG), and multifocal ERG (mERG). During the study period, BCVA, ETDRS and OCT were assessed prior each intravitreal injection. Fluorescein angiography (FA), and indocyanine green angiography (IOG) were performed prior to randomization.

### Full-field electroretinography

FfERGs were recorded with an Espion E^2^ analysis system (Diagnosys, Lowell, Massachusetts, USA) according to the standardized protocol for clinical electroretinography recommended by the International Society for Clinical Electrophysiology of Vision (ISCEV) [11] with slight modifications (the dark-adapted 10 ERG was not recorded). Measurements were recorded with a Burian-Allen bipolar corneal ERG contact lens electrode after 40 min of dark adaptation and with maximally dilated pupils (cyclopentolate 1% and 10% phenylephrine hydrochloride). The ground electrode was placed on the forehead. Responses were obtained with a wide-band filter (− 3 dB at 1 Hz and 500 Hz). To elicit the isolated rod responses, the dark-adapted 0.01 ERG was applied. A brighter white light (3 cd·s/m^2^) also during dark adaptation, was used to measure the combined rod-cone responses (dark-adapted 3. 0 ERG). The isolated cone responses were recorded using 30 Hz flickering white light averaged over 20 sweeps both without and with background illumination (luminance 30 cd/m^2^). Concerning the ffERG parameters, the a-wave amplitude, measured from the baseline to the bottom of the through, is considered to reflect photoreceptor activity [12, 13] while the b-wave amplitude, measured from the bottom of the through to the top of the peak is considered to correspond to bipolar—and Müller cell activity [14–16] and also indirectly photoreceptor function. To ensure reproducibility, the recordings were repeated for all stimulus intensities until two successive identical curves were obtained.

### Multifocal electroretinography

MERGs were recorded with a Visual Evoked Response Imaging System (VERIS Science 6; EDI, San Mateo, USA) using settings that adhere to the ISCEV guidelines [17]. The stimulus matrix consisted of 103 hexagonal elements, scaled with eccentricity to elicit approximately equal amplitude responses at all locations. Each hexagon independently alternated between black and white according to a pseudorandom binary m-sequence at 75 Hz. The pupils were maximally dilated with cyclopentolate 1% and 10% phenylephrine. Retinal activity was registered using a Burian-Allen bipolar ERG contact lens electrode that was placed on the anesthetized (oxybuprocaine) cornea. Fixation was monitored with an infra-red (IR) eye camera, which is built into the equipment. The first order component of the mERG was analyzed regarding amplitudes (A) and implicit times (IT) of P1 (first positive peak) within the concentric rings (A 1–5 and IT 1–5) around the fovea. Ring 1, the innermost ring, represents the summed responses from the central hexagon and the hexagons of the first ring (A 1 and IT 1). The total area of the summed responses represents the central 25° while the innermost seven hexagons forming ring 1 corresponds to 5°.

### Statistical analysis

The data were analyzed using SPSS, version 25.0 (IBM SPSS Statistics, IBM Corporation, Chicago. IL, USA). We applied nonparametic statistical analyses because the group sizes were small, and ERG data are often not normally distributed. The comparison of the mean values between two related samples or measurements was performed by applying the nonparametric Wilcoxon signed-rank test. The Mann–Whitney U-test for nonparametric data was used to compare data from two independent groups. In cases of multiple comparisons of nonparametric data, we used the Kruskal–Wallis test. All tests were two-sided, and a *p*-value of < 0.05 was considered statistically significant. The values are expressed as mean ± standard deviation. The sample size for this pilot study was estimated to be 20 consecutive patients in each arm. Since it was a pilot study, we were not able to do a full power calculation.

## Results

### Baseline characteristics

There was no statistically significant difference in mean baseline visual acuity, lesion type, age, gender, or symptom duration between the two arms. Arm 1 presented with a minimally classic lesion in two eyes (9.5%), a predominantly classic lesion in four eyes (19.0%), an occult lesion in 11 eyes (52.4%), and a RAP in four eyes (19.0%). Arm 2 presented with a minimally classic lesion in two eyes (10.0%), a predominantly classic lesion in seven eyes (35.0%), an occult lesion in 9 eyes (45.0%), and a RAP lesion in two eyes (10.0%). The mean age in arm 1 was 80.3 ± 5.8 years, and 76.4 ± 8.9 years in arm 2. The gender distribution of the total of 41 patients was 14 (66.7%) women in arm 1 vs. 15 (75%) in arm 2. The mean symptom duration was 10.6 ± 7.6 weeks in arm 1, and 10.4 ± 8.1 weeks in arm 2.

### Visual acuity

**Fig. 1:**
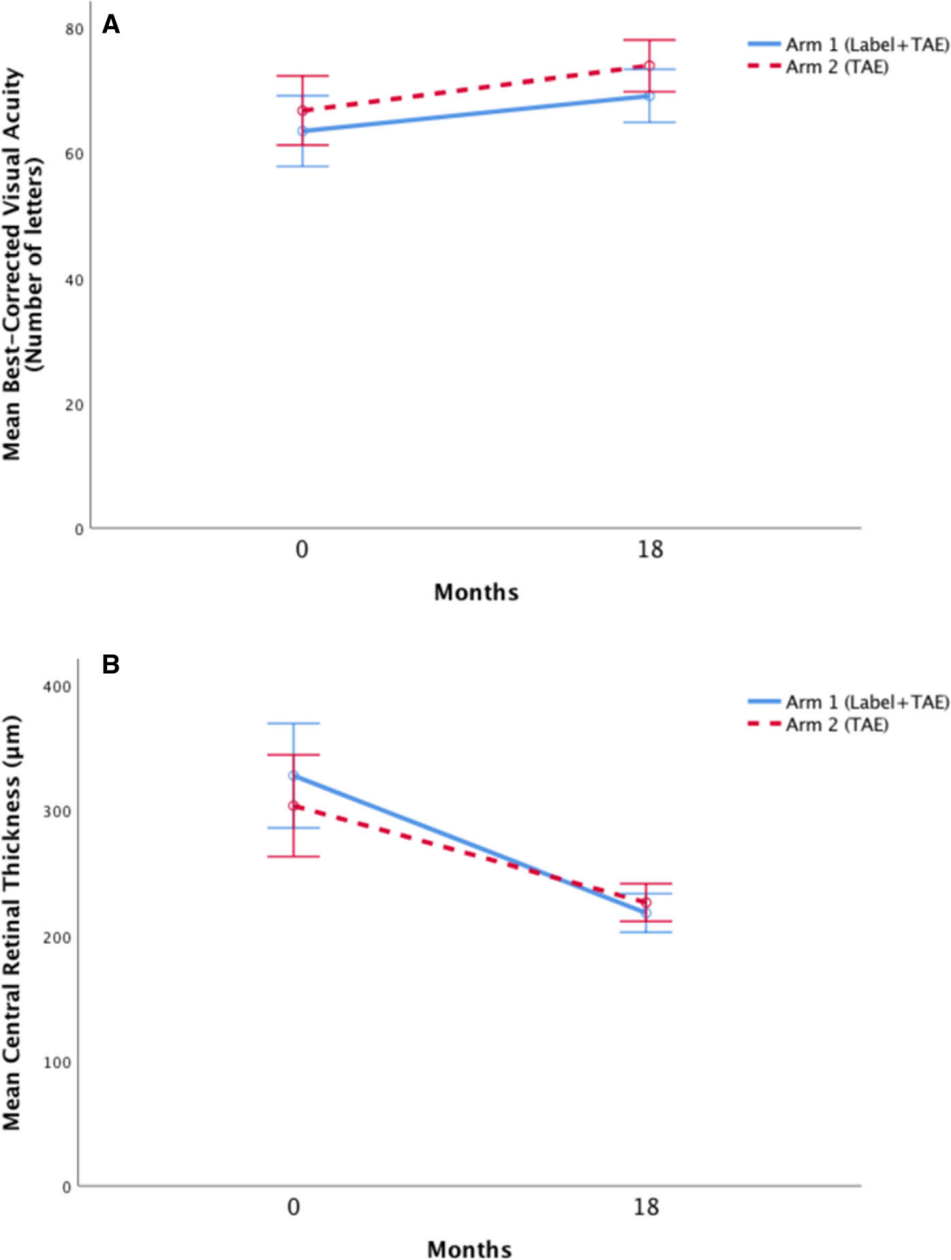
Mean change in visual acuity (**A**) and central retinal thickness (**B**) in treatment arms 1 and 2 from baseline to the final follow-up at 18 months. Error bars represent the 95% confidence intervals (*TAE*  treat-and-extend)

Baseline visual acuity (VA) improved in arm 1 (*n* = 19) by a mean of 5.6 ± 13.3 letters from 63.5 ± 10.5 letters to 69.1 ± 9.2 letters; *p* = 0.098. In arm 2 (*n* = 20), VA increased by a mean of 7.2 ± 8.0 letters from 66.8 ± 13.6 letters to 73.9 ± 9.0 letters; *p* = 0.002. (Fig. 1A).

### Optical coherence tomography

During the 18-month follow-up, mean central retinal thickness (CRT) decreased by 109.7 ± 82.2 μm in arm 1 from 327.8 ± 87.6 μm to 218.1 ± 34.4 μm; *p* < 0.000. In arm 2, CRT decreased by a mean of 77.3 ± 82.5 μm from 303.7 ± 92.0 μm to 226.4 ± 32.3 μm; *p* < 0.000. (Fig. 1B).

### Injections

At 6-month follow-up, patients in arm 1 (*n* = 19) had received a mean of 5.0 ± 0.2 injections compared to 4.7 ± 0.5 injections in arm 2 (*n* = 20); *p* = 0.023. After 18 months, patients in arm 1 had received a mean of 11.3 ± 1.7 injections vs. 10.9 ± 2.0 injections in arm 2 [not significant (NS)].

**Fig. 2:**
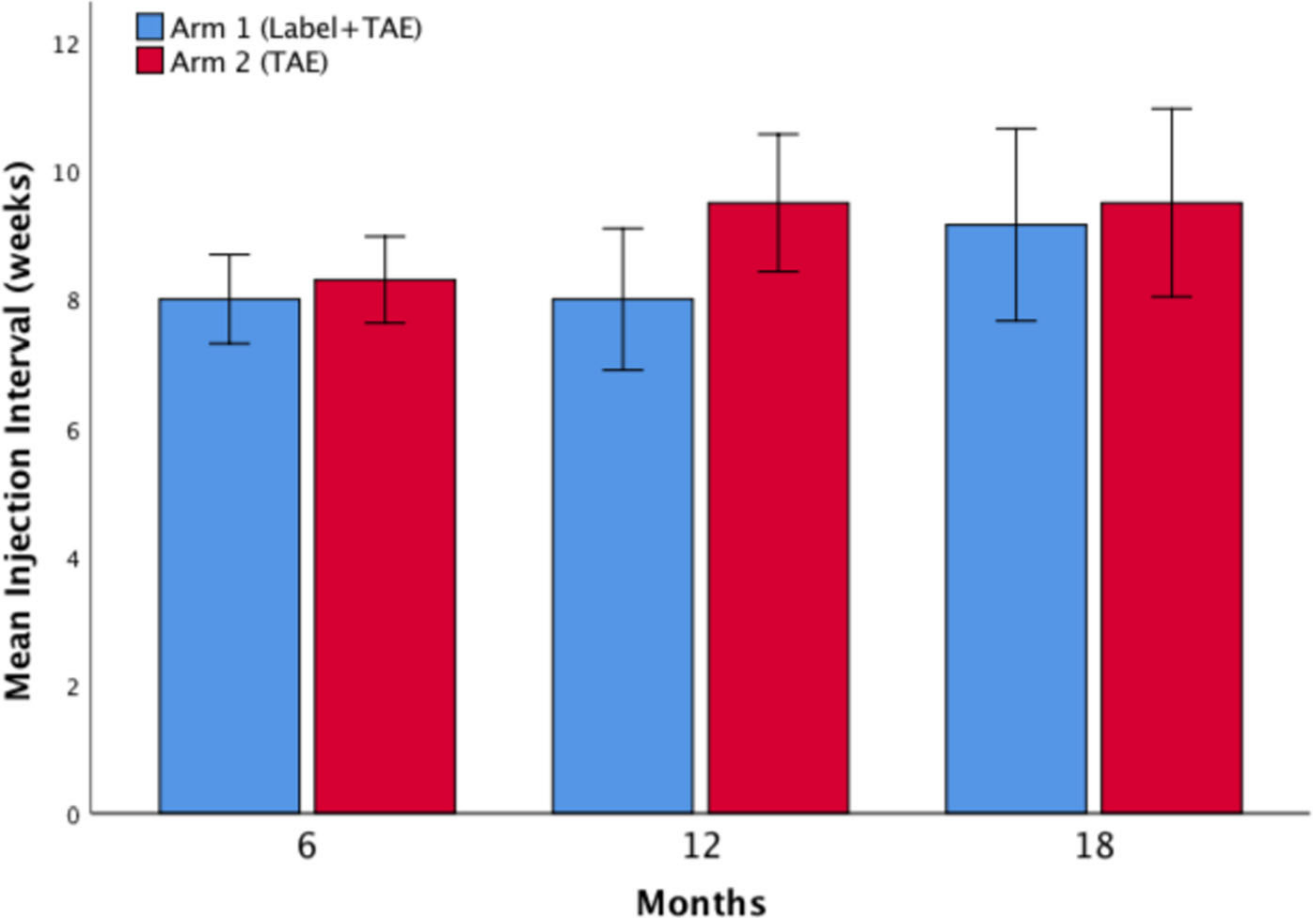
Mean injection interval in treatment arms 1 and 2 at month 6, 12, and 18. Error bars represent the 95% confidence intervals (*TAE* treat-and-extend)

After 6 months of treatment, the injection interval had reached a mean of 8.0 ± 0.0 weeks for patients in arm 1 and 8.3 ± 2.1 weeks for patients in arm 2 (NS). At 12 months, the next planned injection interval was 8.0 ± 2.3 weeks in arm 1 vs. 9.5 ± 2.4 weeks in arm 2; *p* = 0.041. At 18 months, the interval had reached 9.2 ± 3.4 weeks vs. 9.5 ± 3.1 weeks (NS). (Fig. 2) At month 18, the latest injection interval per treatment arm, divided up into 4- to 12-week intervals, is presented in Table 1. The maximum reached mean injection interval was 10.2 ± 2.0 weeks in the arm 1 group vs. 10.5 ± 2.1 weeks in the arm 2 group, during the 18-month follow-up period (NS).

**Table 1:** Showing the mean final injection interval per treatment arm

Final Injection Interval (at month 18)	Arm 1 (Label + TAE)	Arm 2 (TAE)	*p*-value
Mean injection interval (weeks ± SD)	9.2 ± 3.4	9.5 ± 3.1	ns
*Individual intervals*			
4-week interval (*n*; %)	3 (16)	3 (15)	
6-week interval (*n*; %)	4 (21)	2 (10)	
8-week interval (*n*; %)	1 (5)	2 (10)	
10 week interval (*n*; %)	1 (5)	3 (15)	
12 week interval (*n*; %)	10 (52)	10 (50)	

It lists the possible final individual injection intervals, describes the number of patients (*n*), and the corresponding percentage (%) in arm 1 and arm 2. There is no statistically significant difference between the two arms (*SD*  standard deviation; *TAE*  treat-and-extend; *ns*  not significant)

### Full-field electroretinography

Full-field electroretinography (ffERG) represents rod and cone photoreceptor responses from the entire retina. We compared baseline and 18-month results and found no statistically significant differences in the ffERG results concerning the light-adapted isolated 30-Hz flicker cone amplitude and implicit time. The combined rod-cone a-wave amplitude and the isolated rod b-wave amplitude significantly decreased over time; *p* = 0.043 and *p* = 0.026, respectively (Table 2).

**Table 2:** Showing means and standard deviations (SDs) for ffERG amplitudes, implicit times (IT), best corrected visual acuity (BCVA), central retinal thickness (CRT), difference Δ, and *p*-values for the Wilcoxon Signed Ranks Test at baseline and at 18-month follow-up of 39 eyes

	Rod response b-wave (Ampl)	Combined response a-wave (Ampl)	30-Hz flicker Cone response (LA) b-wave (Ampl)	30-Hz flicker Cone response (LA) b-wave (IT)	BCVA (letters)	CRT (µm)
Baseline	201.8 ± 64.0	154.6 ± 53.8	57.9 ± 19.3	30.9 ± 2.4	65.1 ± 12.1	315.4 ± 89.5
18-month follow-up	175.7 ± 75.0	138.0 ± 49.0	54.7 ± 21.9	31.5 ± 2.5	71.5 ± 9.2	222.3 ± 33.1
Difference Δ	26.2 ± 63.3	16.6 ± 47.7	3.2 ± 13.3	.6 ± 2.6	6.4 ± 10.7	93.0 ± 82.8
*p-value Wilcoxon Signed Ranks Test*	*0.026*	*0.043*	*0.171*	*0.105*	*0.001*	*0.000*

It compares the response of the rod b-wave, the combined rod-cone a-wave, and the isolated 30-Hz flicker cone b-wave (*LA* light-adapted)

*p*-value < 0.05 considered to be statistically significant

### Multifocal electroretinography

Multifocal electroretinography measures the cone photoreceptor response of the central retina. The summed mERG amplitude, and the summed mERG implicit time, showed almost identical values at baseline and at 18 months; *p* = 0.878 vs. *p* = 0.922. The central ring 1 mERG amplitude improved slightly over 18 months; *p* = 0.041, with an almost identical implicit time. Ring 2–5 mERG amplitudes and implicit times showed no significant change (Table 3).

**Table 3:** Showing means and standard deviations (SDs) for mERG amplitudes and implicit times (IT) in rings 1–5 and combined ring 1–5 (R1–R5) at baseline and at an 18-month follow-up visit of 39 eyes, additionally, best corrected visual acuity (BCVA), central retinal thickness (CRT), difference Δ as well as the *p*-values for the Wilcoxon Signed Ranks Test

	Ring 1 (Ampl)	Ring 2 (Ampl)	Ring 3 (Ampl)	Ring 4 (Ampl)	Ring 5 (Ampl)	Ring 1 (IT)	Ring 2 (IT)
Baseline	17.5 ± 8.7	15.6 ± 6.7	13.6 ± 5.9	12.7 ± 5.3	13.4 ± 5.8	33.7 ± 5.7	32.4 ± 4.0
18-month follow-up	20.9 ± 11.3	16.1 ± 7.7	13.8 ± 6.6	12.6 ± 6.5	13.7 ± 8.1	33.7 ± 4.1	32.7 ± 3.0
Difference	3.4 ± 10.4	0.5 ± 7.3	0.2 ± 6.6	0.1 ± 6.9	0.3 ± 8.0	0.1 ± 7.4	0.3 ± 4.1
Δ *p-value Wilcoxon Signed RanksTest*	*0.041*	*0.732*	*0.738*	*0.889*	*0.946*	*0.402*	*0.775*

	Ring 3 (IT)	Ring 4 (IT)	Ring 5 (IT)	Combined R1–R5 (Ampl)	Combined R1–R5 (IT)	BCVA (letters)	CRT (µm)
Baseline	31.5 ± 3.4	31.2 ± 3.1	31.6 ± 3.3	19.0 ± 8.2	31.7 ± 3.3	65.1 ± 12.1	315.4 ± 89.5
18-month follow-up	31.6 ± 2.4	31.3 ± 2.3	31.6 ± 2.2	19.7 ± 10.8	31.6 ± 2.09	71.5 ± 9.2	222.3 ± 33.1
Difference	0.1 ± 3.4	0.1 ± 3.2	0.0 ± 3.2	0.6 ± 10.9	0.1 ± 3.2	6.4 ± 10.7	93.0 ± 82.8
Δ *p-value Wilcoxon Signed RanksTest*	*0.994*	*0.729*	*0.827*	*0.878*	*0.922*	*0.001*	*0.000*

*p*-value < 0.05 considered to be statistically significant

**Fig. 3:**
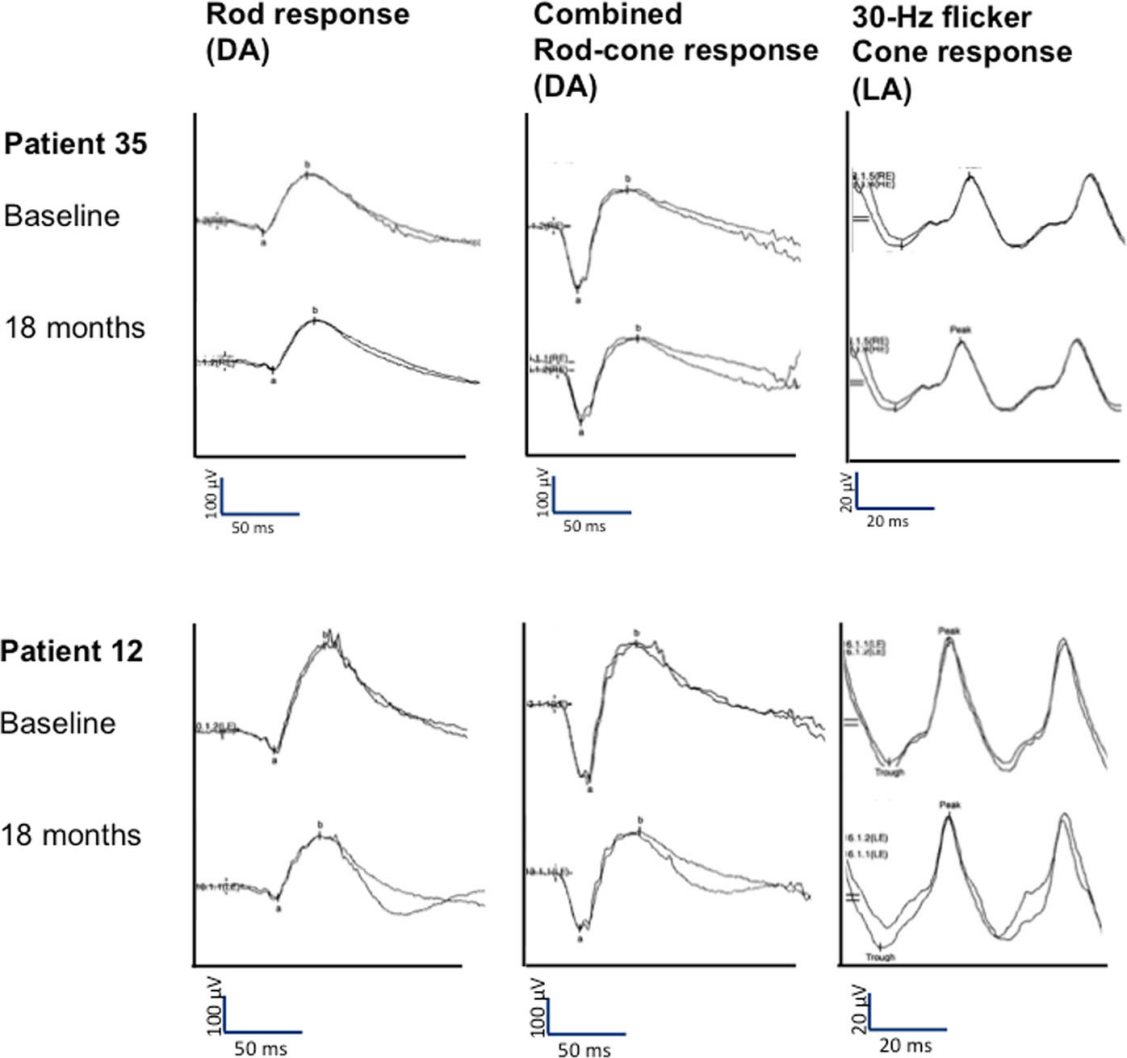
FfERG responses of one representative patient from arm 1 (patient 35) and arm 2 (patient 12), at baseline and at 18 months follow-up. Showing the dark-adapted rod response, the dark-adapted combined rod-cone response, and the light-adapted 30-Hz flicker cone response (*LA* light-adapted)

**Fig. 4:**
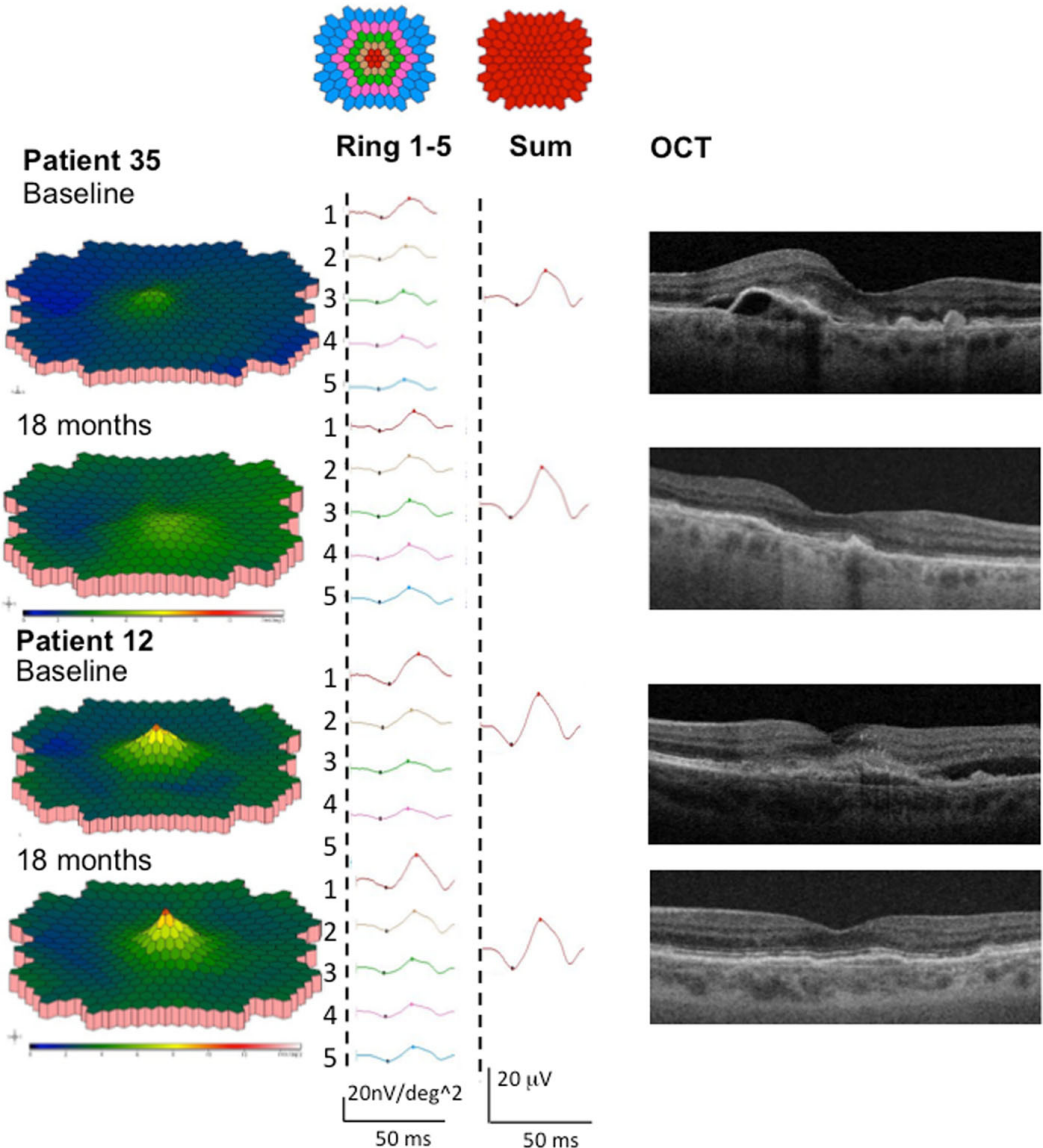
MERG responses and OCT images of one representative patient from arm 1 (patient 35) and arm 2 (patient 12) at baseline and at 18 months follow-up. The 3-D response density plots, the summed mERG response for each ring (ring 1–5) and all rings combined (sum), and the central macula region on OCT (left to right) are shown

Figures 3 and 4 illustrate ffERG, mERG, and OCT images of one representative patient from each treatment arm, at baseline and at 18-month follow-up.

VA and OCT data showed no strong statistically significant correlation to ERG parameters.

## Discussion

**Fig. 5:**
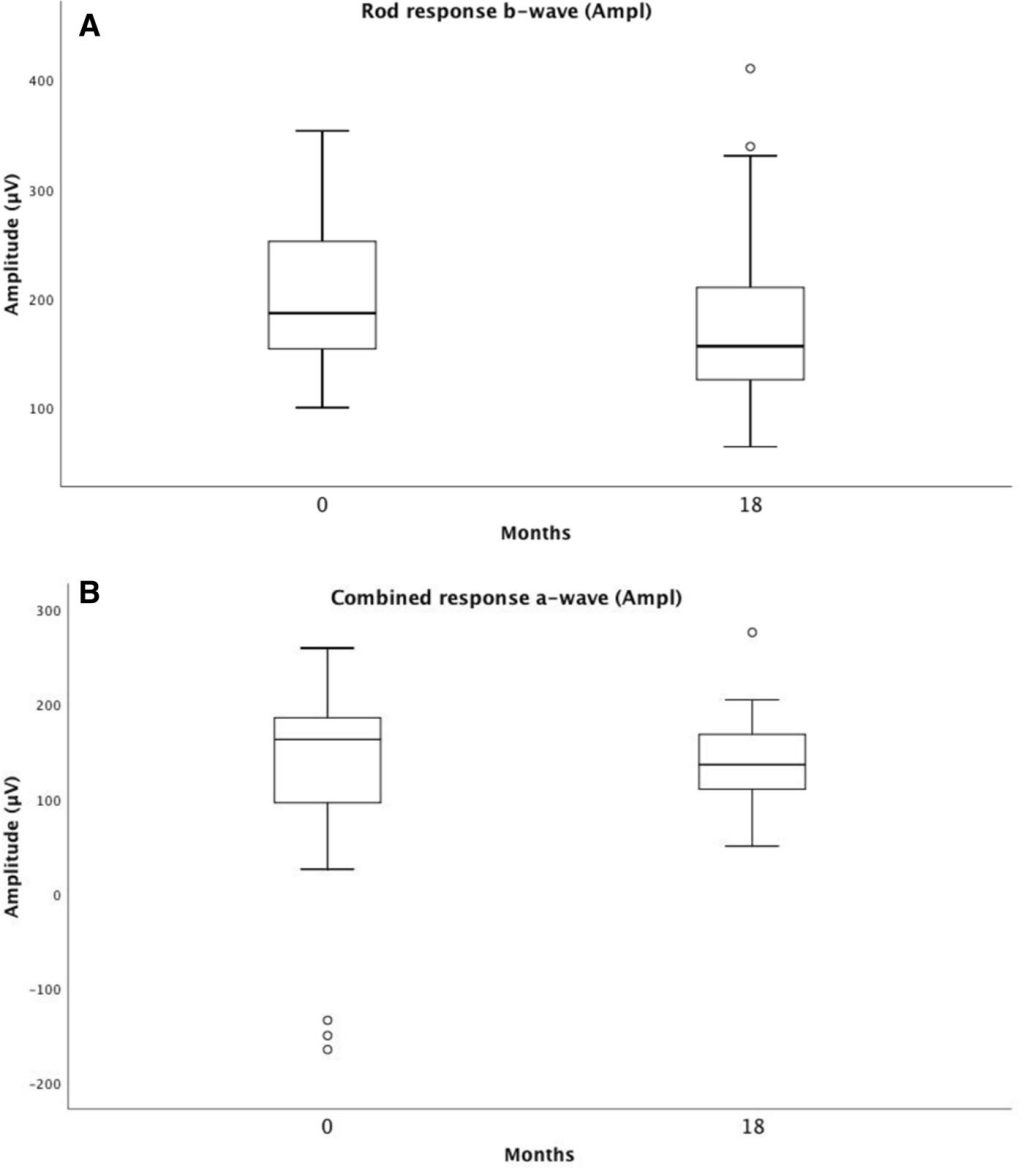

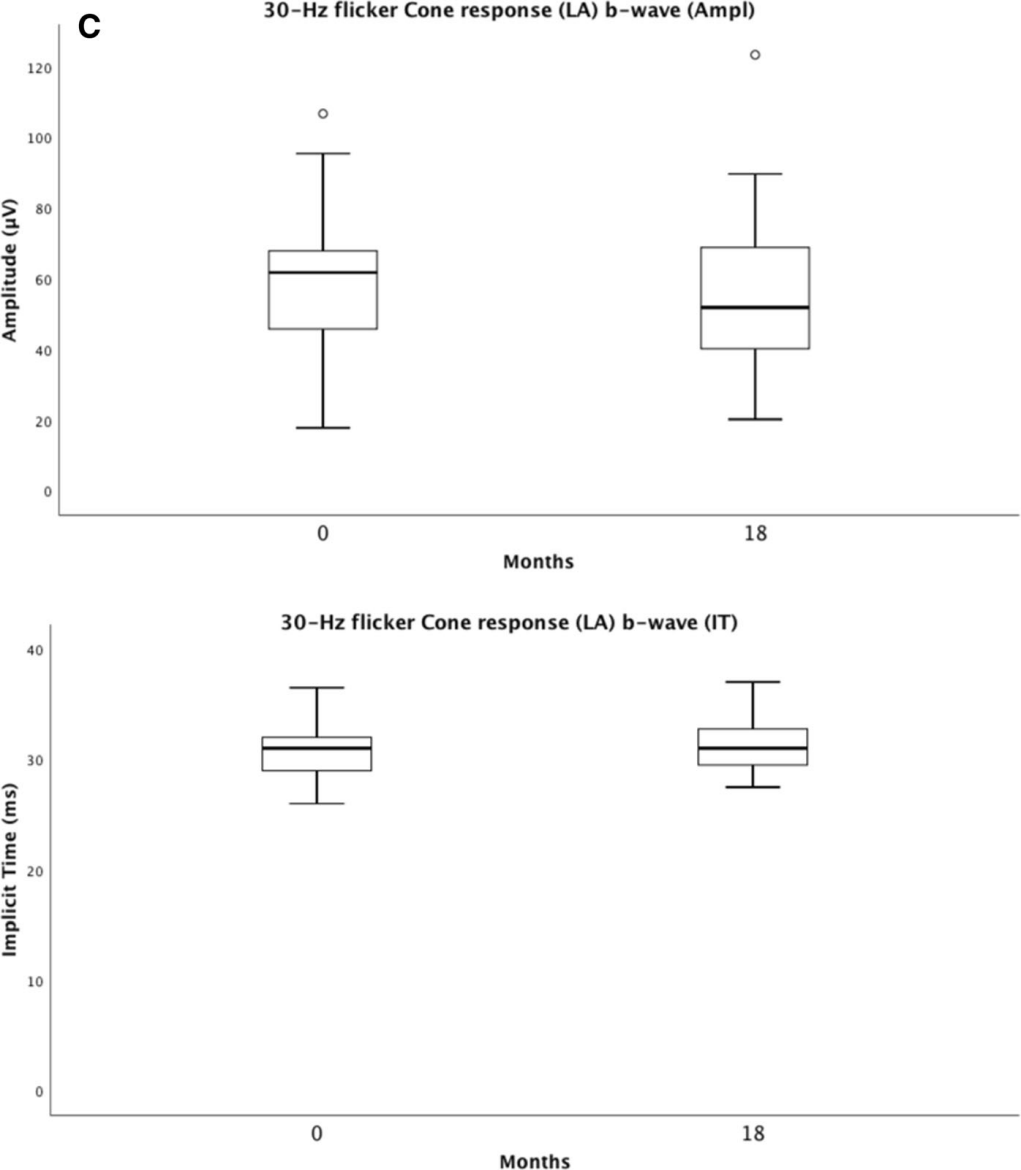
Visualizes the combined ffERG data from baseline (0 months) and the last follow-up (18 months). It shows the inter quartile range and median of the response of the rod b-wave (**A**), the combined rod-cone a-wave (**B**), and the isolated 30-Hz flicker cone b-wave (**C**) with amplitudes and implicit times (IT) (*LA*  light-adapted)

**Fig. 6:**
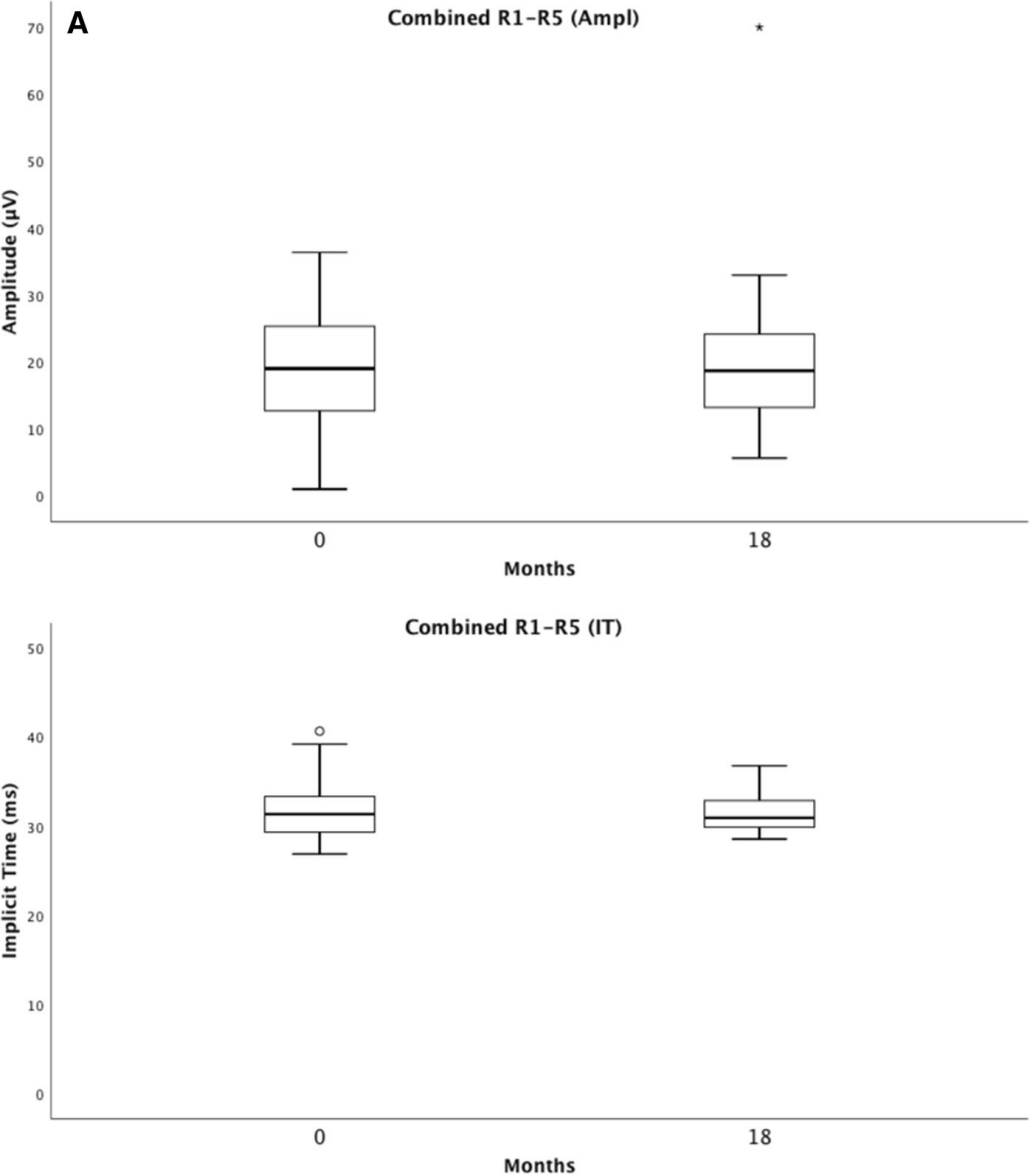

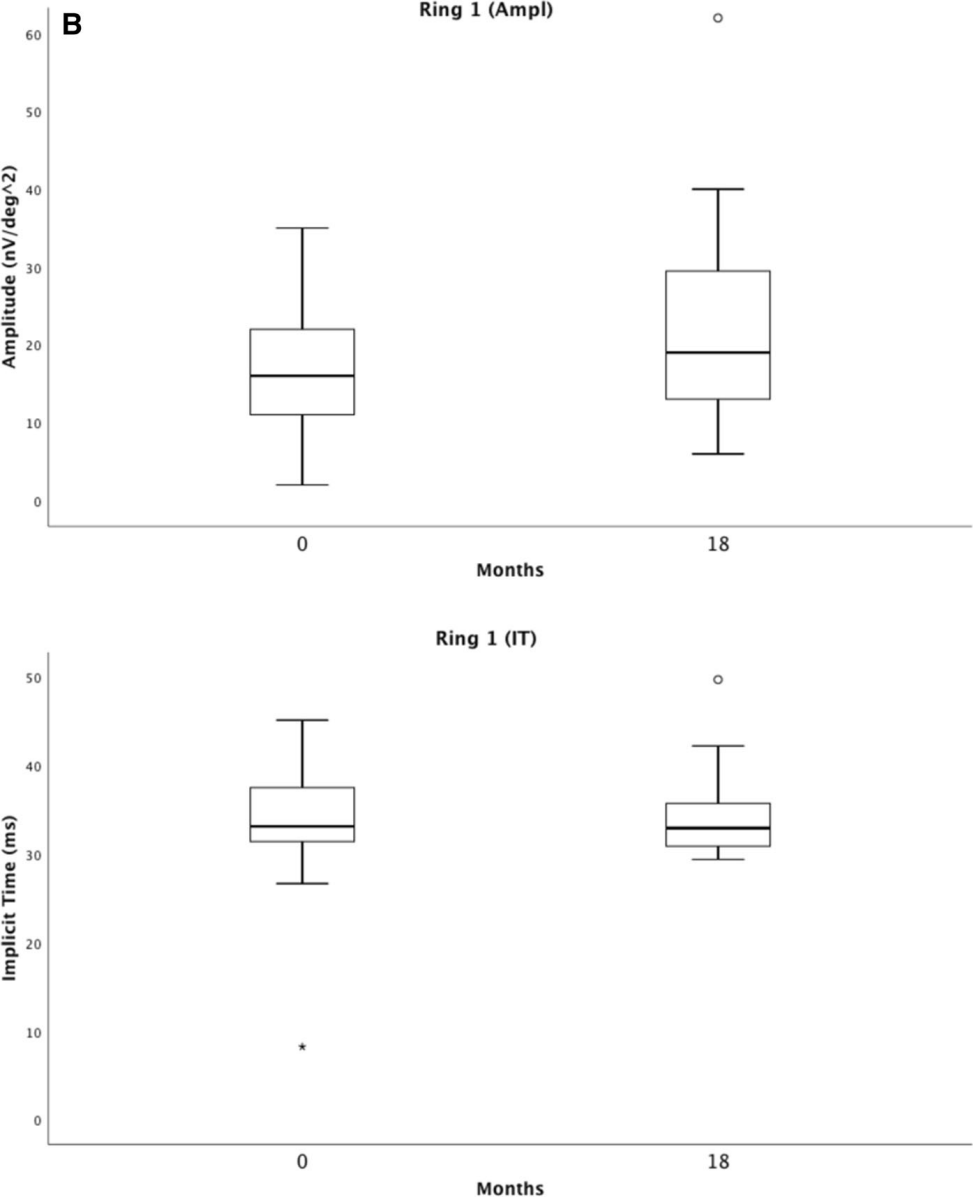
Visualizes the combined mERG data from baseline (0 months) and the last follow-up (18 months). It shows the inter quartile range and median for mERG amplitudes and implicit times (IT) for the combined rings 1–5 (R1–R5) (**A**), and for ring 1 (**B**)

For the entire group of patients in arm 1 and arm 2, ffERG selective cone responses and mERG results are stable during the 18-month follow-up, thus showing no signs of toxic effects on cones in the macula or retinal periphery, Figs. 5 and 6. Our study also demonstrates a slight improvement in the mERG amplitude for the innermost inner, ring 1, but no significant change in the amplitudes of rings 2–5. The implicit times for all 5 rings remain unchanged. This is interesting and probably due to the effect of anti-VEGF on the fovea, leading to a less active CNV. In parity with this, a study in four patients with subfoveal lesions, three with nAMD and one with myopic CNV, followed for 15 months, conducted prior to the anti-VEGF era by Jurklies et al. [18], could demonstrate that mERG findings stabilized and increased in response density corresponding to a decrease in subfoveal CNV activity. In case of increasing CNV activity, a reduction of retinal response densities was encountered. In spite of this, they concluded that the size of the CNV lesion did not exactly reflect retinal function.

In the literature, mERG results after anti-VEGF treatment have shown opposing results. MERG results, similar to the outcomes of the present study, have been published previously for other anti-VEGF agents and treatment regimens, and with a shorter follow-up time; after one or three injections of either bevacizumab or ranibizumab [19–21], after 6 months with consecutive ranibizumab, bevacizumab or both drugs [6, 7, 22], and as a 1-year evaluation of continuous treatment with ranibizumab [23, 24]. Yet another 12-month study has used a bimonthly aflibercept regimen comparable to arm 1 in the present study [25]. Consistent for those studies are mainly improved or unchanged mERG amplitudes and implicit times corroborating our results.

In contrast, other studies have shown a decline in mERG values, maybe due to a different study design with evaluation after only three consecutive ranibizumab injections and with the inclusion of just three patients [26], or eyes with more advanced mAMD with a very low visual acuity of ≤ 20/100 [27].

To our knowledge, there have been only a few publications about electrophysiological assessment of aflibercept in nAMD. Takayama et al. [28] have retrospectively assessed the functional and morphological changes in 42 eyes of 42 treatment-naive nAMD patients who had received three monthly aflibercept injections. Macular function was followed by using focal macular electroretinograms and showed statistically significant improved a- and b-wave amplitudes and implicit times after the third injection compared to baseline. Nishimura et al. [25] have assessed cone function during bimonthly aflibercept treatment of 44 eyes of 44 patients with nAMD at baseline, at three, six and 12 months. They measured the central 15° of the macula with focal macular ERG and reported significantly improvements of all components amplitudes and implicit times at 3 months, some continued to improve, up to the final 12-month follow-up. The majority of their study population consisted of 26 eyes with polypoidal choroidal vasculopathy that were excluded in our study. De Oliveira Dias et al. [29, 30] have published 26- and 52-week nAMD treatment results and data on the safety and efficacy of off-label use of intravitreal ziv-aflibercept, which has been approved for intravenous treatment of metastatic colorectal carcinoma. It is an identical fusion protein to aflibercept with a different buffer solution and purification process. The higher osmolarity might more likely cause retinal toxicity. In the study, mERG amplitudes improved in the central 10° and 5° of the retina by months 6 and 12, correlating to the results of our study. Implicit times remained unchanged after 6 months and improved for P_1_ in the central retinal 5° after 12 months compared to baseline.

Due to different study designs, i.e., concerning what parts of the macula that were investigated, various electrophysiological measurement techniques, different drugs or varying follow-up periods that were applied, it is not possible to directly compare the described studies with our results.

In addition to measurements of macular function with mERG, we also recorded total retinal function using ffERG. The obtained ffERG light-adapted 30-Hz flicker cone responses show stable amplitudes and implicit times over time, which corresponds to the mERG findings (Tables 2 and 3). On the other hand, we see a statistically significant decrease in the combined rod-cone a-wave amplitude for the ffERG where rods dominate the response. An even larger and more significant decline develops for the isolated rod b-wave amplitude during the observation period. This might be due to the natural course of aging and age-related macular degeneration or night vision symptoms in AMD [9, 31, 32]. Unfortunately, since we do not have a control group of nAMD patients that have not received treatment, our data cannot provide fully conclusive evidence of the cause of reduced rod function.

In the literature, rod and cone responses have been described to decline exponentially with age [31]. Other studies have shown age-related reduced amplitudes of the ffERG [33–35]. Weleber et al. [34] have published data showing a 25% loss of ffERG b-wave amplitude from 30 to 67 years of age. Moreover, Dimopoulos et al. [8] have compared dark adaption in eyes with neovascular, dry or no AMD of age-matched subjects showing rod dysfunction in all wet and dry AMD eyes, independently of disease severity. Patients with wet AMD in one eye and dry AMD in the other have presented with rod dysfunction in both eyes.

Another explanation for the generally decreased rod function measured by ffERG could be drug toxicity. In simulated in vivo conditions, bovine retinas have shown significant reduction in a- and b-wave amplitudes directly after exposure to 0.5 and 2 mg VEGF Trap-Eye, aflibercept [5]. At the end of exposure and wash-out time, however, no reduction compared to baseline measurements could be detected. Short-term animal studies have not found retinal toxicity with ERG in the retina of rabbits with aflibercept at 4-week or ziv-aflibercept at 2-week follow-up [36, 37]. Six-month and 1-year follow-up of 15 eyes of nAMD patients treated with ziv-aflibercept have not shown signs of toxicity on ffERG [29, 30].

Other anti-VEGF agents have showed more varying results in rabbits. Cardiakidis Myers et al. [38] have evaluated retinal function after intravitreal bevacizumab, ranibizumab, or pegaptanib injection in comparison with balanced saline solution. FfERG revealed lower amplitudes of the b-waves for the dark-adapted responses to dim light in the anti-VEGF groups. In contrast, Shahar et al. [39] have evaluated rabbits one month after one bevacizumab injection without affected ffERG responses.

Concerning ffERG evaluation in humans treated with anti-VEGF drugs, a study with bevacizumab and shorter follow-up time has also demonstrated an impact on rod function. Pedersen et al. [6] have followed bevacizumab-treated nAMD patients with ffERG for 6 months. Two of three ffERG combined rod-cone responses showed reduction by month 6. However, two other short-term bevacizumab nAMD studies have found stable ffERG responses [40] and improved cone and rod function [41]. Neither of the latter studies have detected any signs of short-term photoreceptor toxicity.

In contrast to our findings, Nishimura et al. [25] have detected a decline of cone function with full-field cone ERG, but without measuring the rod function. They have followed bimonthly aflibercept-treated nAMD patients for 12 months and seen a decreased peripheral cone function, in contrast to improved cone function in the central 15° of the macula. Their full-field cone ERG results have been obtained using another method and another background, they have used a non-standard red stimulus to elicit the cone responses, compared to the presented study. Even their stimulation time differs with 2 ms compared to our values with 20 microseconds. Therefore, these results are not directly comparable.

The described variation in cone and rod function might be due to different AMD phenotypes as proposed by Dimopoulos et al. [8] or peripheral lesions due to nAMD (Tan 2013) which we have not differantiated. Perhaps, in the future it will be possible to differentiate AMD types with the help of electrophysiological evaluations that can uniquely predict the response to future treatments.

The two compared treatment arms show similar baseline characteristics, final mean injection interval, and number of injections at 18-month follow-up. Despite the similarities, the VA increase is statistically significant only in arm 2. A possible explanation for this is that the interval has been more quickly adjusted to an individualized treatment approach in arm 2 than in arm 1. In accordance with the Rival study [42], we did not find any disadvantage in starting intravitreal treatment of nAMD with a treat-and-extend regimen already from the beginning. Our two study arms present with a considerably higher baseline and final BCVA than the pivotal studies for ranibizumab [43, 44] and aflibercept [3], with their baseline mean VA ranging from 47 to 56 letters and final VA from 60 to 66 letters.

The trend toward a higher baseline VA and maintaining visual acuity is probably due to earlier detection, as well as more active and persistent treatment. However, due to the better baseline VA, we experience a ceiling effect with a lower letter gain over time than in patients with lower baseline VA, something that has been demonstrated in some recent studies [45, 46].

Our data, regarding baseline VA and visual outcome, is comparable to the Rival study interim outcome at month 12 [42]. The Rival study has compared the TAE regimen with ranibizumab (baseline VA: 65.3 ± 15.10; final VA: 72.9 ± 15.54 letters) vs. aflibercept (baseline VA: 65.1 ± 12.53; final VA: 70.5 ± 14.63 letters) [42].

Other studies have compared the TAE regimen with monthly ranibizumab. In the TAE arms, patients have gained 6.2 letters with 8.7 injections by the end of year one in the TREND study [47], with +8.4 letters and 9.4 injections slightly more in the CANTREAT study [48], and +10.5 letters with 10.1 injections at the 12-month evaluation in the TREX-AMD study [49]. 24-month data from CANTREAT and TREX-AMD have shown some decrease of visual gain, +6.8 and +8.7 letters, and number of injections, 17.6 and 18.6 injections, compared to the 12-month data [50, 51]. Our 18-month evaluation falls in between the evaluation visits of the other studies with a 7.2 letter gain with 10.9 injections in the TAE arm. Despite, our study presents with a lower number of injections, which might be due to a longer effect of aflibercept. Over the 18-month period, we see a maximum mean injection interval of 10.5 weeks in the TAE group. The TREX-AMD study has described a maximum extension interval of only 8.4 weeks by year one, and 8.5 weeks by the end of year two [49, 51].

A limitation of our study is that we do not have age-matched control ERG material to definitively differentiate a possible toxicity or confirm a progressive rod function decline due to the natural course of AMD. Another shortcoming is that we did not measure retinal function in the fellow eye, which could have provided an estimate of the natural course of change in retinal function over time in these patients.

On the other hand, a strength of this study is that it is, to our knowledge, the largest of its kind to follow-up not only morphological but also functional changes measured by mERG and ffERG over a longer period, and in patients with two different aflibercept treatment regimens.

In conclusion, the primary purpose of our study was to evaluate the effect of intravitreal aflibercept treatment in nAMD on electrophysiological retinal function. The results revealed stable cone function both in the macula and in the periphery without signs of toxicity. On the other hand, rod function was reduced after 18 months, which we could not conclusively explain and might be due to the natural course of AMD or an effect of aflibercept. Thus, further studies with an age-matched control group with nAMD are warranted.

Secondly, we have looked at further aspects of the common intravitreal treatment process for nAMD. We find similar treatment outcomes, number of injections and injection intervals when comparing label vs. TAE treatment regimens for aflibercept over 18 months. This provides further evidence that the already widely used TAE regimen with aflibercept seems to be non-inferior to the bimonthly treatment according to label.
